# Protocol to Assess the Biological Activity of Insulin Glargine, Insulin Lispro, and Insulin Aspart In Vitro

**DOI:** 10.3390/mps6020033

**Published:** 2023-03-24

**Authors:** Mamatha Garige, Susmita Ghosh, Brian Roelofs, V. Ashutosh Rao, Carole Sourbier

**Affiliations:** Office of Biotechnology Products, Office of Pharmaceutical Quality, Center for Drug Evaluation and Research, US Food and Drug Administration, Silver Spring, MD 20993, USA

**Keywords:** in-cell western, potency, insulin, insulin receptor, tyrosine phosphorylation, fluorescence

## Abstract

Insulin is a hormone produced by β-cells of the pancreas and controls the amount of sugar in the blood. Since its discovery over 100 years ago, insulin has been used as a life-saving treatment for people with diabetes. Historically, the biological activity or bioidentity of insulin products has been assessed using an in vivo model. However, reduction in animal experiments is a goal for many worldwide, and there is a need to develop in vitro bioassays to reliably test the biological activity of insulin products. This article describes an in vitro cell-based method to assess the biological activity of insulin glargine, insulin aspart, and insulin lispro in a step-by-step manner.

## 1. Introduction

Insulin, a key regulator of glucose metabolism, is a 51 amino-acid peptide hormone containing two chains: a 21 amino acid A chain and a 30 amino acid B chain, covalently linked via disulfide bridges [1,2]. Insulin is stored in the β-cells of the pancreas as hexamers stabilized by zinc ions. Once in the bloodstream, hexamers are disassembled into monomers, the active form of insulin [3]. At a cellular level, insulin binds to the insulin receptors and the insulin-like growth receptors. In most tissues, insulin binding induces the activation of its receptors by auto-phosphorylation on tyrosine residues, which leads to the tyrosine phosphorylation of multiple downstream targets responsible for the physiological actions of insulin such as glucose uptake, inhibition of adipose triglyceride breakdown, and regulation of energy metabolism [4].

Diabetes is a chronic, life-threatening disease in which the body either does not make enough insulin or has developed insulin resistance [5]. Insulin products have played a very important role in the management of this disease since the discovery of insulin in 1921 by Banting, Best, Collip, and Macleod [6]. Insulin was initially extracted from the pancreas of cattle and pigs before genetically engineered human insulin was produced in 1978 and commercialized in 1982 [7]. Since, various types of insulins with different kinetic profiles have been developed using genetic engineering to address the needs of different populations of patients [8]. Insulin analogs such as insulin glargine, insulin lispro, and insulin aspart differ due to modifications of a few amino acids: for insulin glargine, asparagine ^A21^ has been changed to glycine ^A21^ and has two additional arginine residues in B30 and B31; for insulin lispro, proline ^B28^ has been changed to lysine ^B28^ and lysine ^B29^ to proline ^B29^; and, for insulin aspart, proline ^B28^ has been changed to aspartate ^B28^ [9]. The time-action or kinetic profile of commercial human insulins and insulin analogs depends on multiple factors, such as the excipients contained in their formulation, the amino acid sequence, post-translational modifications, and their pH. As such, insulin glargine is a long-acting insulin (sometimes referred to as a basal insulin) and starts having an effect 1–2 h after injection; insulin aspart and insulin lispro are rapid-acting insulins, they start working less than 15 min after injection and peak between 30 and 90 min [10]. These differences across products create the need for significant assay optimization and, sometimes, lengthy experiments when performed in vivo. In an in vitro cell-based assay, commercial insulins and reference materials, such as the USP standards, are highly diluted during sample preparation leading to the formation of insulin monomers in the diluted samples. Hence, an in vitro cell based assay is amenable to be extended to multiple insulin analogs, regardless of their physiological time-action profiles, with minimal protocol adaptation.

To monitor and control the quality of insulin products, the assessment of both their potency and biological activity, or bioidentity, is an established scientific and regulatory expectation. The potency of well-established insulins has been historically inferred from content, which is commonly assessed by RP-HPLC, and US Pharmacopeia (USP) compendial methods are available for several insulin analogs. The bioidentity of insulin products has been historically assessed using a compendial in vivo test in rabbits where the biological activity of insulin is compared to the effects of a USP reference standard. The USP chapter <121> [11], which describes testing of bioidentity for insulin products, was updated in December 2020 with an in vitro cell-based bioidentity test, an in-cell western assay for insulin glargine and insulin lispro. Reduction in animal experiments, especially for batch release testing, is a worldwide goal for many, and there is an emphasis on transferring historical in vivo bioassays into functional in vitro bioassays [12,13]. Multiple groups have already published in vitro bioassays to assess the biological activity of insulin products [14,15,16,17,18]. For example, Sommerfeld and collaborators described the in vitro bioassay in a subclone of Chinese Hamster Ovary cells (CHO-K1) overexpressing the insulin receptor B for insulin glargine that was included in the USP chapter <121> [11,17]; Yie and collaborators described the development and validation of a functional cell-based assay in rat hepatoma cells (H4-II-E) for insulin glargine using a Glucose-6-phosphatase (G6P)-Luc reporter [16]; and Goyal and collaborators used several AlphaScreen assays in human hepatoma (HepG2) cells or in CHO-K1 cells overexpressing the insulin receptor A or B to compare the potency of insulin glargine with a proposed biosimilar [15].

Possessing in-house the capability of a cell-based insulin potency assay could aid both drug development and regulatory science while offering robust alternatives to animal-based testing. Therefore, we optimized and validated an in-cell western assay in our laboratory, modifying a few steps from the USP <121> protocol for insulin glargine and insulin lispro, such as using fluorescent reagents that could be detected by broadly accessible plate readers instead of the reagents suggested in USP <121> that are detectable at 700 nm and 800 nm. We also developed and validated a protocol for testing the bioidentity of insulin aspart. The protocols for in vitro bioidentity testing of insulin glargine, insulin lispro, and insulin aspart are described in this manuscript.

## 2. Experimental Design

### 2.1. Materials

The catalog numbers listed below were used in our method development, but equivalent material may also be suitable upon validation.

1. Bovine Serum Albumin (Sigma, St. Louis, MO, USA, #A9647);
2. 96-well microplate, black, polystyrene (Sigma, #CLS3603);
3. 96 well plates, polypropylene (Sigma, #M9685-100EA);
4. Accutase (Sigma, #A6964-100ML);
5. 37% (*w*/*v*) formaldehyde solution (Sigma, #252549-25ML);
6. Hygromycin B (Invitrogen, Waltham, MA, USA, #10687010);
7. Ham’s F-12 Nutrient Mix with GlutaMAX (Invitrogen, #31765092);
8. Fetal Bovine Serum, certified, heat-inactivated (Invitrogen, #A3840002);
9. PBS, no calcium, no magnesium (Invitrogen, #14190250);
10. CHO INSR 1284 (ATCC, Manassas, VA, USA, CRL-3307™);
11. F-12K Medium (ATCC, #30-2004);
12. Hoechst 33342 (Thermo Fisher, Waltham, MA, USA, #62249);
13. Goat anti-Mouse IgG (H + L) secondary antibody, Alexa Fluor 488 (Invitrogen, #A28175);
14. 75 cm^2^ cell culture flask (GSS, Reston, VA, USA, #CLS430641U-100EA);
15. Triton X-100 (GSS, #1086432500);
16. Polysorbate 20 (GSS, #1547925-2G);
17. Dimethyl sulfoxide (GSS, #D2438-5X10ML);
18. Anti-phosphotyrosine antibody (GSS, #05-321);
19. USP Insulin Aspart (7.62 mg) (USP, Rockville, MD, USA, #1342037);
20. USP Insulin Glargine (15.06 mg) (USP, #1342059);
21. USP Insulin Lispro (5.73 mg) (USP, #1342321).

### 2.2. Equipment

1. A plate reader with the capability to read:
  o Alexa Fluorophore 488: Ex/Em: 493/519
  o Hoechst 33342: Ex/Em: 361/486

We used an EnSight or an EnVision multimode plate reader (Perkin Elmer);

2. Access to a biosafety cabinet that adheres to institutional BSL2 requirements to perform sterile cell culture.

### 2.3. Experimental Design including the Time Needed to Complete Every Stage

The molecular basis of this assay is the biological effect of insulin when it binds to the insulin receptor. A commercially available CHO-K1 cell line stably over-expressing human insulin receptor was used (CHO INSR 1284, ATCC^®^ CRL-3307™). When insulin binds its receptor, it triggers the auto-phosphorylation of tyrosine residues on the receptor. Quantifying insulin-induced auto-phosphorylation of the insulin receptor is determined as a read-out for insulin and insulin analog biological activity.

**Figure 1:**
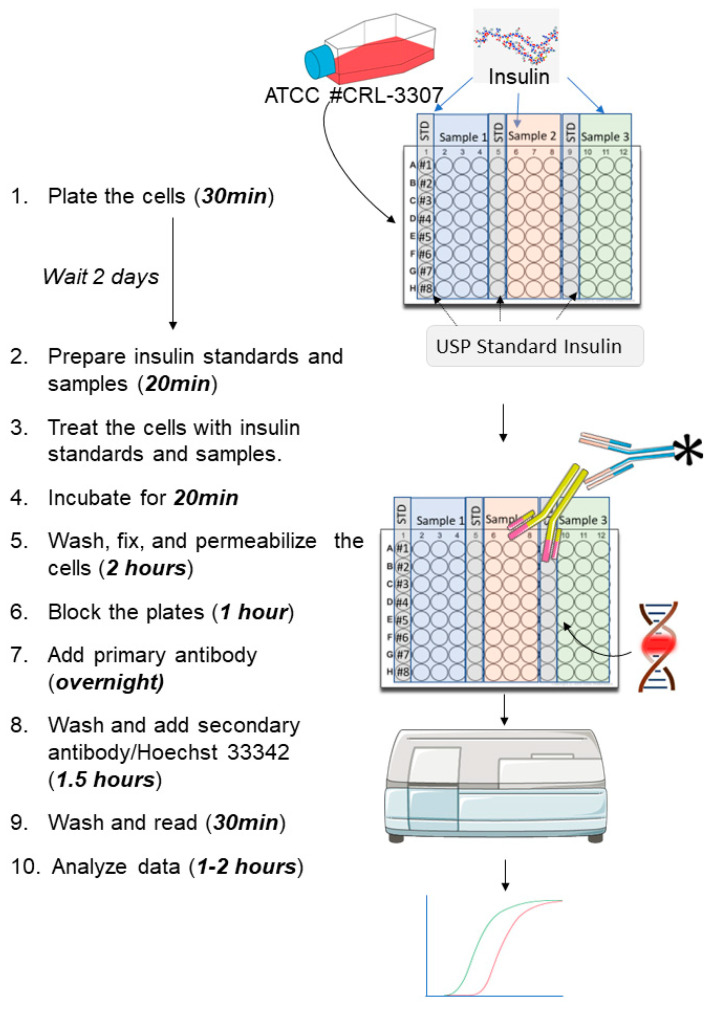
Experimental design of the in-cell western assay.

The phospho-tyrosine residues are then recognized by an anti-phospho-tyrosine primary antibody followed by a secondary antibody tagged with a fluorescent dye. DNA intercalating agent staining is used simultaneously to normalize the results to the cell number. The final detection is performed with a fluorescence plate reader. A depiction of the experimental design, including the time needed to complete every stage, is shown in Figure 1.

## 3. Procedure

### 3.1. Cell Culture

1. Culture CHO INSR 1284 cells in complete medium (Ham’s F-12/Glutamax + 10% (*v*/*v*) FBS, + 0.03% (*v*/*v*) hygromycinB) at 37 °C and 5% carbon dioxide (CO_2_) atmosphere in a humidified incubator;
2. When cells reach 80–95% confluency, remove the flasks from the incubator and discard the media;
3. Rinse the cells one time with 1.5 mL of Accutase;
4. Add 2.5 mL of Accutase to the cells and place the flask in an incubator for about 2–3 min at 37 °C. Tap gently on the flask to detach the cells from the flask’s bottom. Add 5 mL of complete medium to stop the Accutase activity;
5. Determine cell concentration and viability with trypan blue;
6. Calculate the desired number of cells for seeding;
7. Prepare at least two 96-well plates: For each plate, add 0.2 mL of cell solution at 0.9 × 10^5^ viable cells/mL in complete medium per well of a sterile 96-well, clear bottom, black polystyrene plate:
  o Mix the cell solution frequently during dispensing to prevent cells from settling and ensure consistent density throughout the plate;
  o Note: Two plates are necessary per run. One standard and three samples from two independent dilutions can be assessed using 2 plates;
  o OPTIONAL STEP: let the plates stand at room temperature for 30 min before transferring them into an incubator to minimize an edge effect;
8. Cover the plates and incubate them at 37 °C with 5% CO_2_ for 2 days;
9. CRITICAL STEP: Verify homogeneous monolayer growth and 90–95% confluency via microscope before performing the experiment.

### 3.2. Preparation of the Insulin Samples

CRITICAL STEP: USP insulins are reconstituted at a concentration of 100 U/mL in 0.01 N hydrochloric acid (note that the insulin powder is sometimes very close to the inside/sides of the cap. The reconstitution has to be done very carefully to ensure that all material is reconstituted). Preparation of the insulins (standards and samples) and treatment of the cells must occur within 15–20 min of preparation on the day of the experiment. The dilutions are prepared in two 96-well polypropylene plates. The two plates are technical replicates and are used to evaluate the robustness of the experiment, for example, by calculating the relative bias for each plate.

#### 3.2.1. Insulin Glargine

1. Dilute 100 U/mL USP insulin glargine in 0.01 N hydrochloric acid to generate a stock solution of 17 U/mL;
2. Dilute the 17 U/mL standard stock solution to 0.34 U/mL with PBS. Prepare in duplicate for the preparation of the second plate;
3. Dilute insulin samples to 0.34 U/mL in PBS. Prepare in duplicate for the preparation of the second plate;
4. Add 200 μL of PBS solution to rows B-H of a 96-well polypropylene plate;
5. Add 300 μL of standard (0.34 U/mL) into at least three wells of row A of the dilution plate;
6. Add 300 μL of the first insulin sample (0.34 U/mL) into at least three wells of row A of the dilution plate;
7. Add 300 μL of the second sample (if necessary) into at least three wells of row A of the dilution plate;
8. Add 300 μL of the third sample (if necessary) into three wells of row A of the dilution plate;
9. Perform simultaneously serial three-fold dilutions on the plate using a multichannel pipette:
  - Transfer 100 μL of solution from row A to row B;
  - Mix three times with a multichannel pipette;
  - Transfer 100 μL of solution from row B to row C;
  - Mix three times;
  - Repeat this procedure across the whole plate up to row H;
10. Repeat the procedure described above (steps 4–9) for the second plate using the solutions prepared in duplicate (steps 2–3).

**Figure 2:**
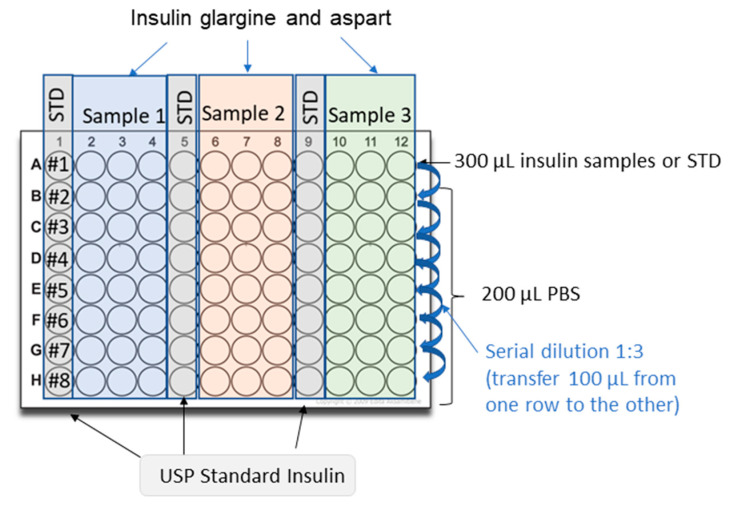
Schematic representation of the preparation of the dilution plates containing insulin glargine or insulin aspart’s standards and samples.

A picture of the setup of the plates to prepare the samples for insulin glargine and aspart is shown in Figure 2.

#### 3.2.2. Insulin Aspart

1. Dilute 100 U/mL USP insulin aspart in PBS to generate a standard stock solution of 17 U/mL;
2. Dilute the 17 U/mL standard stock solution to 0.34 U/mL with PBS. Prepare in duplicate for the preparation of the second plate;
3. Prepare the insulin samples to be tested by diluting them to 0.34 U/mL in PBS. Prepare in duplicate for the preparation of the second plate;
4. Add 200 μL of PBS solution rows B-H of a 96-well polypropylene plate;
5. Add 300 μL of standard (0.34 U/mL) into at least three wells of row A of the dilution plate;
6. Add 300 μL of the first sample (0.34 U/mL) into at least three wells of row A of the dilution plate;
7. Add 300 μL of the second sample (if necessary) into at least three wells of row A of the dilution plate;
8. Add 300 μL of the third sample (if necessary) into three wells of row A of the dilution plate;
9. Perform simultaneously serial three-fold dilutions on the plate using a multichannel pipette:
  - Transfer 100 μL of solution from row A to row B;
  - Mix three times with a multichannel pipette;
  - Transfer 100 μL of solution from row B to row C;
  - Mix three times;
  - Repeat this procedure across the whole plate up to row H;
10. Repeat the procedure described above (steps 4–9) for the second plate of dilution using the solutions prepared in duplicate (steps 2–3).

#### 3.2.3. Insulin Lispro

1. Dilute 100 U/mL USP insulin lispro in 0.1% (*w*/*v*) BSA in PBS to generate a stock solution of 10 U/mL;
2. Dilute the 10 U/mL stock solution to 0.3 U/mL with 0.1% (*w*/*v*) BSA in PBS. Prepare in duplicate for the preparation of the second plate;
3. Dilute insulin samples to 0.3 U/mL in 0.1% (*w*/*v*) BSA in PBS. Prepare in duplicate for the preparation of the second plate;
4. Add 300 μL of 0.1% (*w*/*v*) BSA in PBS to rows D-H of a 96-well polypropylene plate;
5. Add 180 μL of 0.1% (*w*/*v*) BSA in PBS into the wells of rows B and C;
6. Add 300 μL of standard (0.3 U/mL) into at least three wells of row A of the dilution plate;
7. Add 300 μL of the first sample (0.3 U/mL) into at least three wells of row A of the dilution plate;
8. Add 300 μL of the second sample (if necessary) into at least three wells of row A of the dilution plate;
9. Add 300 μL of the third sample (if necessary) into three wells of row A of the dilution plate;
10. Perform simultaneously serial dilutions on the plate using a multichannel pipette:
  - Transfer 120 μL of solution from row A to row B;
  - Mix three times with a multichannel pipette;
  - Transfer 60 μL of solution from row B to row C;
  - Mix three times;
  - Transfer 60 μL of solution from row C to row D;
  - Mix three times;
  - Repeat this procedure across the whole plate up to row H.
11. Repeat the procedure described above (4–10) for the second plate using the solutions prepared in duplicate (steps 2–3).

**Figure 3:**
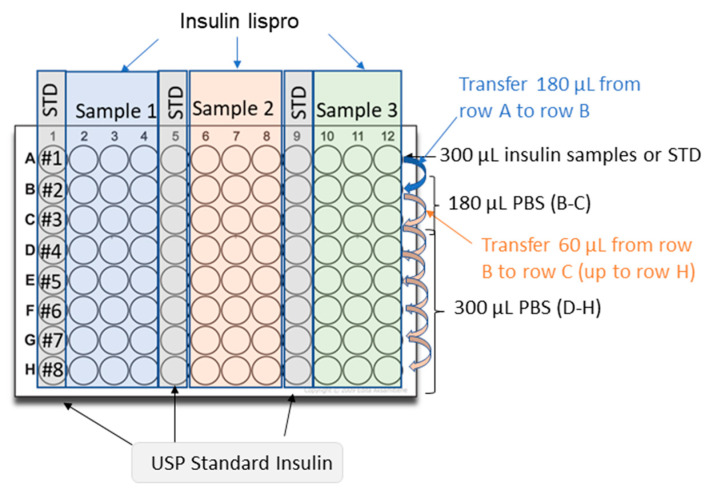
Schematic representation of the preparation of the dilution plates containing insulin lispro’s standards and samples.

A picture of the setup of the plate to prepare the samples for insulin lispro is shown in Figure 3.

### 3.3. Treatment of the Cells and Performing the in-Cell Western

1. On the day of the experiment, remove media from the cells and wash each well once gently with PBS;
2. Add 160 μL of Ham’s F-12/Glutamax (without FBS) to each well. Incubate plates for 3–5 h at 37 °C with 5% CO_2_;
3. Add 40 μL per well of each dilution of insulin standard and test samples into the appropriate wells of the plate;
4. CRITICAL STEP: Incubate the plates at 37 °C with 5% carbon dioxide (CO_2_) for 20 min;
5. Discard the media and wash it one time with PBS;
6. Add 150 μL of 3.7% formaldehyde solution per well (dilute the 37% formaldehyde solution in PBS) to fix the cells. Incubate the plates for 20 min with constant gentle orbital shaking (40–50 rpm);
7. Discard the formaldehyde solution, wash it one time with PBS, and add 200 μL/wells of 0.2% (*v*/*v*) Triton X-100 (diluted in PBS) to permeabilize the cells;
8. Incubate the plates for 10 min with constant gentle orbital shaking (40–50 rpm);
9. Repeat the permeabilization step one more time.
10. Discard the permeabilization solution, and wash 1 time with PBS:
  o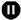 PAUSE STEP: the covered plate containing PBS could be stored at 4 °C overnight;
11. Add 300 μL/well of blocking buffer (2% (*w*/*v*) BSA in PBS);
12. Incubate the plates at room temperature for 1 h;
13. Remove the blocking buffer and add 50 μL/well of primary antibody solution (dilute 1:1000 the anti-phospho-tyrosine mouse monoclonal antibody in 2% (w/v) BSA in PBS supplemented with 0.1% PS20);
14. Cover the plates and incubate them at 2–8 °C overnight with gentle shaking;
15. Discard the primary antibody solution and wash each well three times using 200 μL/well of washing solution (0.1% (*v*/*v*) PS20 in PBS). Perform each wash step under constant gentle shaking for about 7 min;
16. Add 50 μL/well of the secondary antibody and Hoechst solution (dilute 1:1000 the goat anti-mouse secondary antibody conjugated with Alexa Fluor 488 into 2% (*w*/*v*) BSA in PBS supplemented with 0.2% PS20 and Hoechst 33342 at 0.5 µg/mL);
17. Incubate the plates protected from light (use black lids or equivalent) for about 1 h with constant gentle shaking at room temperature;
18. Discard the secondary antibody solution and wash each well three times using 200 μL of washing solution (0.1% (*v*/*v*) PS20 in PBS); perform each wash step under constant gentle orbital shaking for about 7 min;
19. Keep the plates in 200 μL/well of washing solution (0.1% (*v*/*v*) PS20 in PBS), protected from light until measurement:
  o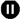 PAUSE STEP: The plates containing the washing solution can be stored at 4 °C for a maximum of 24 h while protected from light;
20. Read the plate in a plate reader with appropriate excitation and emission wavelengths:
  o Alexa Fluorophore 488: Ex/Em: 493/519;
  o Hoechst 33342: Ex/Em: 361/486.

Depending on the plate reader, two separate programs might need to be used (one for each wavelength). Other plate readers may allow for the reads to be done automatically and successively. If two separate programs are needed, try to perform them as fast as possible.

## 4. Results

### 4.1. Analyses of the Data

Using software that allows for the analysis of a four-parameter logistic (4PL) constrained model is recommended to analyze the data.

1. Normalize each well per number of cells using the assay signal detected from the secondary antibody relative to the signal from Hoechst;
2. Subtract the background signal from the normalized signal. The background signal is the average of the normalized signals detected by the diluted standard solution at the lowest concentration of insulin;
3. Generate a 4PL dose-response curve for each standard and sample;
4. The linearity of the curves (R^2^ ≥ 0.95) should be assessed for each curve;
5. Determine EC_50_ for the standard curve and the samples from 4PL dose-response curves;
6. Relative potency is calculated by dividing the EC_50_ from the standard with the EC_50_ from the sample and multiplying by 100. Relative Potency = (EC_50_, standard/EC_50_, sample) × 100;
7. The mean relative potency is calculated by calculating the geometric mean of the relative potency of the two plates that were prepared in parallel;
8. Relative bias (RB) for the relative potency of the samples is calculated for each plate from the relative potency calculated from each plate and the mean relative potency. RB plate x = [(relative potency plate x/mean relative potency) − 1] × 100;
9. Additional parameters, including relative standard deviation, slope, and parallelism, could be used to ensure the consistency of this assay;
10. Depending on the intended use of this assay, a full validation may be required to confirm that the assay is suitable for the intended purpose with predefined acceptance criteria for successful application.

### 4.2. Potential Results

**Figure 4:**
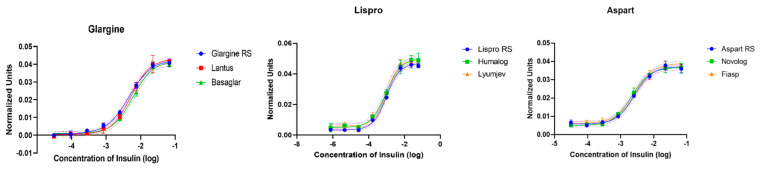
S-shape sigmoidal curves obtained after 4PL modeling. Normalized data are used to generate 4PL dose-response curves for the US reference standards and the different insulin samples. “RS” refers to USP reference standards.

Representative curves of the potency of six insulin products related to their USP reference standards are shown in Figure 4. Commercial insulin products were purchased via McKesson Specialty Health (Scottsdale, AZ, USA), and USP reference standards were purchased from USP. Because we tested only two samples, four replicates of each sample were tested in each plate in parallel to their respective reference standard. Insulin glargine analogs Lantus (Sanofi) and Basaglar (Eli Lilly and Company) were compared to the USP reference standard insulin glargine; insulin lispro analogs Humalog (Eli Lilly and Company) and Lyumjev (Eli Lilly and Company) were compared to the USP reference standard insulin lispro; and insulin aspart analogs Novolog (Novo Nordisk) and Fiasp (Novo Nordisk) were compared to the USP reference standard insulin aspart. The assays described in this protocol for these three insulin analogs were validated in our laboratory. Briefly, 18,000 cells/wells were plated into two black-well plates for each of the analogs. Cells were grown for 2 days. As indicated in the protocol, insulin standards and samples were prepared and used to treat cells for 20 min. After washing, fixing, and permeabilizing, the cells were stained, and the fluorescent signal was detected using a multimodal plate reader (EnSight, Perkin Elmer). Results were exported from the instrument as excel spreadsheets, and data were normalized as described in Section 4.1. The normalized data were then transferred into GraphPad Prism to generate the 4PL dose-response curves. All the curves, including those for the reference standards, passed R^2^ ≥ 0.95; therefore, further analysis was performed.

Results of the relative potency assessment for the six insulin products by in-cell western potency assays are shown in Table 1. All the tested samples had a geometric mean of relative potency between 90% and 105%, CV ≤ 12%, and relative bias ≤ 9. Based on our validation studies, these values are within the acceptance criteria of our assay, and all the samples meet their potency requirements.

## 5. Conclusions

Insulin researchers and manufacturers have developed in vitro potency assays to replace or complement the traditional in vivo rabbit blood sugar method for a few insulin analogs. The protocol described in this manuscript describes another option for researchers to easily assess the biological activity of insulin glargine, aspart, or lispro in vitro.

## Figures and Tables

**Table 1:** Examples of in-cell western insulin bioassay results for insulin glargine, insulin lispro, and insulin aspart.

Values in Comparison to USP Reference Standards	Relative Potency (RP, %)		Mean Relative Potency (MU, %)	% CV	Relative Bias (RB)	
Insulin glargine	Plate A	Plate B			Plate A	Plate B
Lantus	90.22	104.26	97.24	10.21	−6.97	7.49
Basaglar	104.61	95.54	100.07	6.41	4.64	−4.43
Insulin lispro	Plate A	Plate B			Plate A	Plate B
Humalog	91.69	108.1	99.89	11.60	−7.89	8.57
Lyumjev	95.94	84.98	90.46	8.57	6.26	−5.88
Insulin aspart	Plate A	Plate B			Plate A	Plate B
Novolog	112.12	95.91	104.01	11.46	8.12	−7.51
Fiasp	105.61	96.5	101.05	6.44	4.614	−4.41

## Data Availability

Not applicable.
